# Dnmt3a2 expression during embryonic development is required for phenotypic stability

**DOI:** 10.21203/rs.3.rs-6908216/v1

**Published:** 2025-07-01

**Authors:** Peter Jones, Minmin Liu, Guillermo Urrutia, Rachel Shereda, Stacey Thomas, Gangning Liang

**Affiliations:** Van Andel Institute; Van Andel Institute; Van Andel Institute; University of Southern California

## Abstract

Proper function and switching of regulatory elements are essential for the development of vertebrates and is known to be controlled by DNA methylation. We used isoform-specific knockouts of the *de novo* methyltransferase Dnmt3a, namely Dnmt3a1 and Dnmt3a2, to probe their roles in regulatory element methylation during embryogenesis and postnatal development. Mouse embryos lacking Dnmt3a1 showed minimal loss of methylation, suggesting limited involvement in embryonic development. However, they were smaller than their littermates and died about 4 weeks after birth with considerable postnatal demethylation as previously reported. In contrast, embryos lacking Dnmt3a2 showed widespread hypomethylation particularly at enhancers, CTCF sites and imprinted genes. These methylation deficits were largely repaired after birth, presumably by Dnmt3a1. The mice lacking Dnmt3a2 were viable; however, they showed an increased prevalence of sporadic abnormalities previously observed at a low frequency in laboratory mice, including anophthalmia, hydrocephalus, hydronephrosis and male infertility. Interestingly, hypomethylation of several imprinted genes was observed in sperm which might explain the infertility phenotype. Therefore, the interaction between the two isoforms is developmentally regulated, with Dnmt3a2 playing a key role in ensuring the methylation states of enhancers, CTCF sites and imprinted genes, thereby reducing the likelihood of stochastic phenotypes emerging after birth.

## Introduction

DNA methylation is an epigenetic modification that plays a critical role in regulation of gene expression by modulating the activity of key regulatory elements such as promoters, enhancers, CCCTC-binding factor (CTCF) binding sites and Imprinting control regions (ICRs)^1^. These regulatory elements mediate spatial and temporal control of gene expression, ensuring precise transcriptional activation during development^2,3^. The control of gene expression is fundamental to proper formation and function of tissues and organs, as it dictates tissue-specific gene expression patterns^4,5^. However, there is still limited *in vivo* evidence linking impaired DNA methylation machinery to regulatory element dysfunction particularly for enhancer and imprinted genes, which could ultimately lead to developmental abnormalities.

DNA methylation involves the addition of a methyl group to the 5′ position of cytosine residues within CpG dinucleotides, and is mainly established by the *de novo* DNA methyltransferases, including Dnmt3a and Dnmt3b, during development in mammals^6^. Mutations in *DNMT3A* have been implicated in a spectrum of human diseases, particularly hematological malignancies^7–11^ and developmental disorders, including Tatton-Brown-Rahman syndrome (TBRS)^12^ and microcephalic dwarfism^13^. These findings underscore the important role of *DNMT3A* in normal development and its contribution to disease when dysregulated.

Existing total and tissue-specific Dnmt3a KO mouse models have revealed diverse and context-dependent phenotypes, highlighting the importance of Dnmt3a in various biological processes. For example, homozygous Dnmt3a KO mice (*Dnmt3a*^−/−^) display severe developmental defects, including growth retardation, premature death, and impaired germ cell development^6^. In contrast, heterozygous Dnmt3a KO mice (*Dnmt3a*^+/−^) exhibit postnatal phenotypes such as obesity, increased bone length, and behavioral abnormalities, which partially recapitulate human TBRS caused by DNMT3A mutations^14,15^. Tissue-specific KO models further demonstrate that Dnmt3a is essential for germ cell development^16^, hematopoietic stem cell (HSC) differentiation^17^, neurogenesis^18,19^, and suppression of tumorigenesis in a conditional mouse lung tumor model^20^. However, most studies investigating Dnmt3a function using mouse models have not distinguished between its two major isoforms, Dnmt3a1 and Dnmt3a2, which are generated through alternative promoter usage.

Dnmt3a1 features a 219-amino-acid-long N-terminal region, which is missing in Dnmt3a2, while the rest of the protein sequences are the same for both isoforms. The two isoforms exhibit distinct expression patterns, regulatory mechanisms, and potentially unique biological functions^21^. For example, while Dnmt3a1 is ubiquitously expressed and thought to be the predominant isoform in most somatic tissues, Dnmt3a2 is highly expressed in embryonic stem cells (ESCs) and during early development, suggesting it may have specialized roles in maintaining pluripotency or regulating rapid developmental transitions^21^. A recent study has reported isoform-specific mouse models and found that Dnmt3a1, but not Dnmt3a2, is essential for postnatal survival^22^. This highlights the role of Dnmt3a1 in the nervous system regulating bivalent neurodevelopmental genes through interactions of its unique N-terminus with monoubiquitinated histone H2AK119^22^. In addition, Dnmt3a2 has been shown to be essential for maintaining genomic imprinting and epigenomic integrity in mouse embryonic cells^23–25^. These isoform-specific studies have begun to enhance our understanding of their distinct roles in development and disease. Despite their distinct expression profiles and potential functional differences, the individual contributions of these isoforms to methylate different regulatory elements during development remain poorly understood.

To dissect the specific roles of Dnmt3a1 and Dnmt3a2 in modulating the methylation of regulatory elements, we generated isoform-specific knockout models to investigate how each isoform contributes to the establishment and maintenance of methylation patterns during both embryonic and postnatal development. This approach provides an opportunity to unravel the different functions of these isoforms in modulating methylation of regulatory elements leading to eventually distinct developmental phenotypes.

## Results

### Dnmt3a2 knockout mice exhibit stochastic abnormalities during embryonic and postnatal development

**Figure 1:**
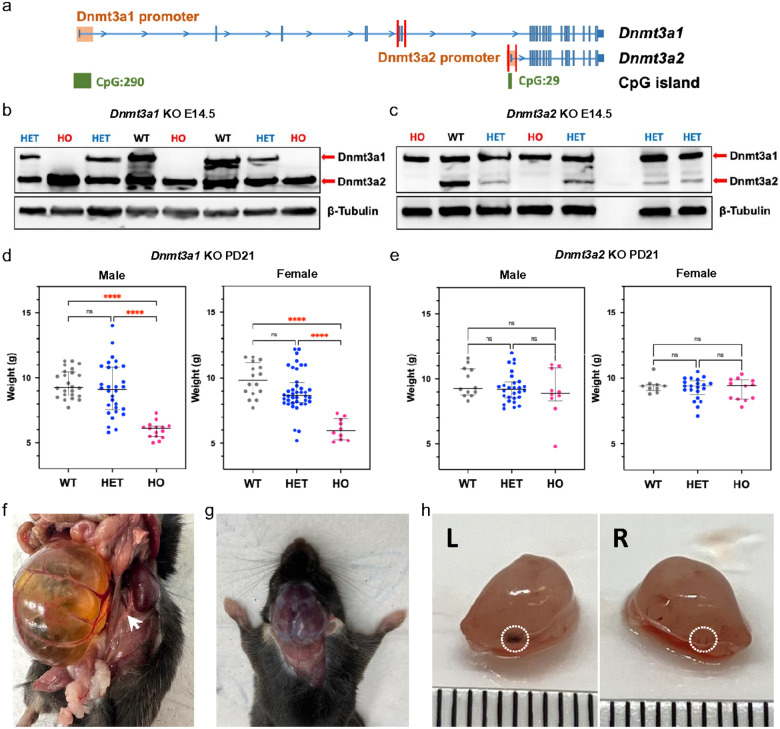
Sporadic phenotypes in *Dnmt3a2*^−/−^ mice. **a,** Diagram illustrating strategies by CRISPR-Cas9 genome editing to generate *Dnmt3a* isoform-specific KO models. The cut sites for each guide RNA were represented by red bars. **b and c,** Western blot analysis of E14.5 mouse embryos show specific KO of Dnmt3a1 (**b**) and Dnmt3a2 (**c**) in homozygous (HO) embryos compared to their heterozygous (HET) and WT littermates. **d and e,** Body weights of *Dnmt3a1 KO* (**d**) and *Dnmt3a2 KO* (**e**) mice at 21 days postnatal. Data shown as median ± 95%CI with individual values; ns, not significant and ****, p < 0.0001 by one-way ANOVA with Tukey’s multiple comparisons test. **f,** Representative image of hydronephrosis in a five-month old *Dnmt3a2*^−/−^ mouse. Note that there is no ureter connecting the right kidney with the bladder. White arrow shows normal ureter on the left side. **g,** Representative image of hydrocephalus in a two-month old *Dnmt3a2*^−/−^ mouse. **h,** Representative image of anophthalmia in an E15.5 *Dnmt3a2*^−/−^embryo. White dotted circle shows the hypoplastic eye on the right side.

To examine the function of the two different Dnmt3a isoforms during development, we generated isoform-specific *Dnmt3a* knock-out mouse models. We used CRISPR-based genome editing to target exons 5 and 6 (744 bp) in mouse zygotes to delete *Dnmt3a1* specifically (Fig. 1a), resulting in frame shift and premature termination during translation (Extended Data Fig. 1). To delete Dnmt3a2, we targeted the promoter and exon1 of *Dnmt3a2* (941 bp), which are located within the intron 6 of the *Dnmt3a1* locus (Fig. 1a). Dnmt3a1 or Dnmt3a2 proteins were completely depleted in the respective E14.5 homozygous knockout embryos without affecting the expression of the other isoform (Fig. 1b, c). As previously reported^22^, the growth of *Dnmt3a12*^−/−^ mice was delayed, as shown by the reduced body weight compared to wildtype (WT) mice at postnatal day 21 (PD21) (Fig. 1d). All Dnmt3a1^−/−^ pups were runted followed by death between 3 and 4 weeks after birth, showing similar phenotypes as the *Dnmt3a*-null mice^6,22^. The body weight of both male and female *Dnmt3a1*^+/−^ mice exhibited a bimodal distribution, with increased variation compared to their WT littermates at PD21 (Fig. 1d), similar to previously reported phenotypes of Dnmt3a^+/−^ mice^26^. The observation that Dnmt3a1 KO mice phenocopied Dnmt3a KO mice suggests that Dnmt3a1 is the predominant isoform and plays a critical role in postnatal development.

Although the male and female *Dnmt3a2*^−/−^ mice were viable and their body weights showed no differences compared to their WT and *Dnmt3a2*^+/−^ littermates at PD21 (Fig. 1e), sporadic Dnmt3a2^−/−^ mice exhibited reduced (50%) bodyweight (4.5%, Fig. 1e). We also observed the incidence of sporadic developmental defects which were manifested from 14.5 days during embryonic development to 6 months postnatal (Fig. 1f–h). These include hydronephrosis due to unilateral ureteral agenesis with a frequency of 5 out of 203 (2.5%) *Dnmt3a2*^−/−^ mice (Fig. 1f and Table 1). The incidence of hydrocephalus was 2 out of 203 (1%) (Fig. 1g and Table 1), characterized by the entire brain being filled with cerebrospinal fluid. True anophthalmia also occurred in 4 out of 203 (2%) *Dnmt3a2*^−/−^ mice, and 1 out of 560 (0.18%) *Dnmt3a2*^+/−^ mice, characterized by the complete absence of one or both ocular structures due to failed optic vesicle formation during early embryogenesis (Fig. 1h and Table 1). In addition, out of 70 actively breeding males, 4 were identified as infertile, exhibiting an inability to successfully sire offspring despite confirmed mating activity, corresponding to an infertility rate of 5.7% within the tested cohort (Table 1). Collectively, the cases for the stochastic developmental defects were over 15% in *Dnmt3a2*^−/−^ mice, which were not found in the WT or *Dnmt3a1*^+/−^ mice (Table 1), although some developmental defects have previously been reported in the heavily inbred C57BL6 background (https://www.informatics.jax.org/inbred_strains/mouse/docs/C57BL.shtml). The observation that knocking out Dnmt3a2 increases the incidence of developmental defects in a stochastic manner suggests a role of the Dnmt3a2 to ensure phenotypic stability during embryonic development.

### Dnmt3a2 participates in de novo methylation during embryonic development

**Figure 2:**
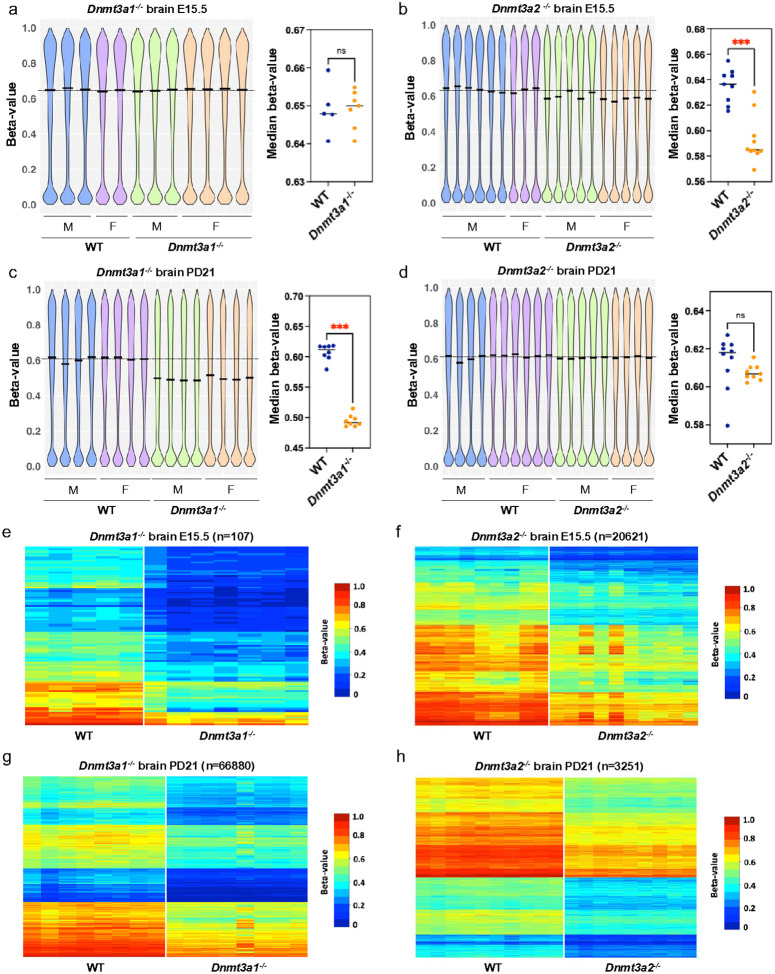
Hypomethylated CpGs in brain from *Dnmt3a1*^−/−^ and *Dnmt3a2*^−/−^ mice during embryonic and postnatal development. **a-d,** Distribution of CpG methylation for the autosomal probes from the MM285 array in brain of the *Dnmt3a1*^−/−^ (**a,c**) and *Dnmt3a2*^−/−^ (**b,d**) mice at E15.5 and PD21. The left panels show violin plots for the distribution of CpG methylation as beta-values in WT and KO brain. Males are represented as M and females are represented as F. The median beta-value in WT brain is marked by the dotted line. The right panels compare the median beta-value for the WT to the KO brain. ns, not significant and ***, p < 0.001 by two-tailed unpaired Mann-Whitney U test. **e-h,** Heatmap representing the hypomethylated CpGs in brain of the *Dnmt3a1*^−/−^ (e,g) and *Dnmt3a2*^−/−^ (**f,h**) mice at E15.5 and PD21. Hypomethylated probes were selected using a cutoff of beta-value difference greater than 0.1 and p < 0.05 by two-tailed unpaired Mann-Whitney U test. Methylation levels are represented by a cold to warm color scale (β-value 0–1, 0 – 100% methylated), where every row represents an individual probe, and every column represents sample from an individual mouse.

We next investigated the DNA methylation changes that might be associated with the stochastic developmental defects observed in *Dnmt3a2*^−/−^ mice compared to their WT litter mates, focusing on two critical developmental windows: E15.5, and 21 days postnatal (PD 21). These time points were selected to capture methylation dynamics during peak embryonic DNA methylation establishment (E15.5) and during postnatal tissue maturation (PD 21). To examine tissue-specific DNA methylation patterns, we isolated brain and liver tissues and used the Illumina MM285 Infinium Methylation EPIC array to perform genome-wide methylation analysis. Methylation values for each probe (CpG site) were expressed as beta values (β-value), defined as the ratio of methylated probe intensity to overall probe intensity^27^. We first examined the overall DNA methylation pattern in both male and female *Dnmt3a1*^−/−^ and *Dnmt3a2*^−/−^ mice compared to their WT littermates by visualizing the distribution of β-values for all autosomal probes. At E15.5, the median levels of DNA methylation for brain and liver in WT mice are 0.64 and 0.50, respectively, showing tissue-specific methylation patterns (Fig. 2a and Extended Data Fig. 2a). While *Dnmt3a1*^−/−^ embryos did not exhibit overall methylation changes (Fig. 2a and Extended Data Fig. 2a), a significant reduction (p < 0.001) of the median DNA methylation levels were observed in *Dnmt3a2*^−/−^ embryos (0.585 and 0.478 for brain and liver, respectively), showing hypomethylation in *Dnmt3a2*^−/−^ embryos (Fig. 2b and Extended Data Fig. 2b). These data indicate that Dnmt3a2 contributes to establish *de novo* methylation during embryonic development, while Dnmt3a1 is dispensable, despite being highly expressed at this stage (Fig. 1b). However, at PD21, substantial hypomethylation (p < 0.001) for *Dnmt3a1*^−/−^ mice was observed (Fig. 2c and Extended Data Fig. 2c), while the overall hypomethylation in *Dnmt3a2*^−/−^ mice was not significant (p = 0.0653 for both brain and liver) (Fig. 2d and Extended Data Fig. 2d). We also used a liquid chromatography-mass spectrometry (LCMS) method to measure the global 5mC level at PD21 and found a significant reduction (p < 0.0001) of 5mC level in the brains of *Dnmt3a1*^−/−^ mice but not in the liver (Extended Data Fig. 3), possibly because site specific methylation patterns may not be captured at the global level.

To further quantify the level of hypomethylation, we used 0.1 β-value difference and p < 0.05 as a cutoff to select the probes that showed consistent hypomethylation in our KO tissues compared to WT (Fig. 2e–h). In *Dnmt3a1*^−/−^ mice, minimal hypomethylation was detected at E15.5 in both brain (0.041%) and liver (0.018%) (Fig. 2e, Extended Data Fig. 2e and Table 2). However, by PD21, the level of hypomethylation escalated dramatically, reaching 26% in brain and 12% in liver, reflecting a profound postnatal decrease in the level of methylation (Fig. 2g, Extended Data Fig. 2g and Table 2). This progressive lack of methylation suggests that Dnmt3a1 is dispensable during early embryonic development for *de novo* methylation but becomes critical for establishing methylation during postnatal maturation. Tissue-specific differences were pronounced, with brain exhibiting double the hypomethylation of liver, underscoring a heightened reliance on Dnmt3a1 in neural tissue for maintaining postnatal epigenetic stability as previously published^22^.

In contrast, *Dnmt3a2*^−/−^ mice displayed distinct embryonic-predominant hypomethylation. Applying the same beta-value cutoff (≥ 0.1 reduction vs. wildtype and p < 0.05), significant embryonic hypomethylation was observed at E15.5 in brain (8.1%) and liver (3.8%) (Fig. 2f, Extended Data Fig. 2f and Table 2), consistent with Dnmt3a2’s role in *de novo* methylation during organogenesis. Postnatally, hypomethylation levels declined to 1.3% in brain and 1.8% in liver by 21 days (Fig. 2h, Extended Data Fig. 2h and Table 2), suggesting partial recovery of DNA methylation by Dnmt3a1 after birth. This temporal reversal implies that Dnmt3a2 is indispensable for establishing methylation patterns during embryogenesis, but its absence is partially mitigated postnatally, likely through compensatory mechanisms. Taken together, these findings suggest that Dnmt3a2 is mainly responsible for establishing *de novo* methylation during embryonic development (E15.5), while Dnmt3a1 plays the dominant role during the postnatal stage (PD21).

### Dnmt3a2 ensures proper de novo methylation especially at enhancers during embryonic development

**Figure 3:**
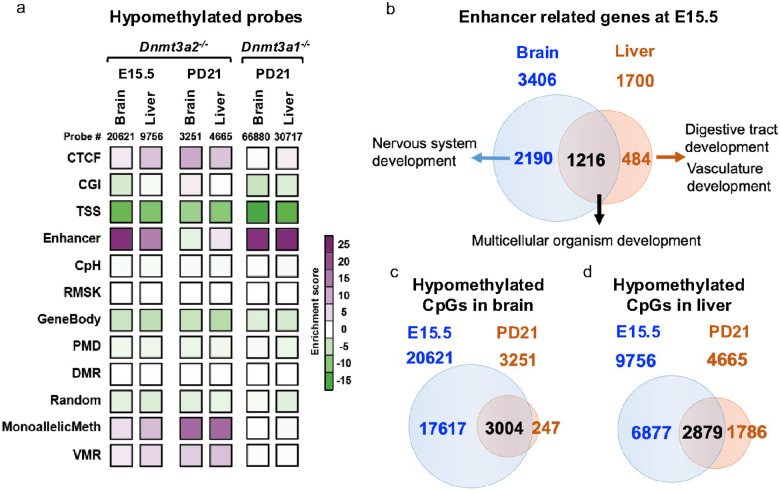
Hypomethylated CpGs in *Dnmt3a2*^−/−^ embryos are enriched at enhancers. **a,** Heatmap represents enrichment of hypomethylated CpGs in brain and liver from *Dnmt3a1*^−/−^ at PD21 and *Dnmt3a2*^−/−^ mice at E15.5 and PD21. The classified genomic elements are as follows: CTCF binding sites (CTCF), CpG island (CGI), transcription start site (TSS), enhancer (Enhancer), CpH methylation (CPH), repetitive elements (RMSK), gene bodies (GeneBody), partially methylated domains (PMD), differentially methylated regions (DMR), random location probes (Random), monoallelic methylation, including the ICRs of imprinted genes (MonoallelicMeth), and metastable alleles and variably methylated regions (VMRs). **b,** Venn diagram shows about half of the hypomethylated enhancers in *Dnmt3a2*^−/−^ embryos are near genes driving tissue specific development. **c and d,** Venn diagrams show in brain (**c**) and liver (**d**), the methylation deficits in embryos were largely repaired after birth.

As we mentioned previously, *Dnmt3a2*^−/−^ mice exhibit stochastic phenotypes during embryonic and postnatal development. We then further investigated the genomic regions where the hypomethylated probes regulated by Dnmt3a2 are located, using classified genomic elements designed in the MM285 array^27^. We then calculated the enrichment of hypomethylated probes in these genomic elements against their overall distribution in the mouse EPIC arrays. We found that the hypomethylated probes were highly enriched in enhancers, CTCF sites and monoallelic methylation regions in Dnmt3a2^−/−^ brain and liver at E15.5 (Fig. 3a). Interestingly, the enrichment for enhancers disappeared mostly at PD21, but the CTCF sites and imprinted genes (MonoallelicMeth) were still enriched (Fig. 3a). In contrast, *Dnmt3a1*^−/−^ brain and liver exhibit hypomethylation highly enriched exclusively for enhancers after birth (Fig. 3a). These results suggest that Dnmt3a2 is important for establishing DNA methylation at enhancers and CTCF binding sites during embryonic and/or postnatal development. In contrast, the observed enrichment of hypomethylation at imprinted genes (MonoallelicMeth) may result from defects in *de novo* methylation establishment in the gametes of the *Dnmt3a2*^+^/^−^ parental mice. Although Dnmt3a2 is important to establish DNA methylation during embryonic development, its activity on enhancers could be fully compensated by the function of Dnmt3a1 after birth, but Dnmt3a2’s activity on CTCF sites and imprinted genes cannot be compensated by Dnmt3a1 after birth.

As previously established, enhancers are tissue-specific, and our findings further demonstrate that Dnmt3a2 regulates enhancer DNA methylation in a tissue-specific manner. At E15.5, the enhancer related genes identified in brain and liver of the *Dnmt3a2*^−/−^ mice were partially overlapped (Fig. 3b). We analyzed the gene ontology (GO) of enhancer-related genes using functional clustering of the GO terms and found that the overlapping genes in brain and liver were largely involved in multicellular organism development processes, while the other half of the hypomethylated enhancers in *Dnmt3a2*^−/−^ embryos are near genes driving tissue specific development (Fig. 3b). Intriguingly, these methylation deficits were largely repaired after birth (Fig. 3c, d), possibly due to the compensatory activity of the remaining DNA methyltransferases, including the Dnmt3a1 isoform. The majority of probes remaining hypomethylated in PD21 *Dnmt3a2*^−/−^ brain and liver were enriched for CTCF sites and imprinted genes (Fig. 3a). This tissue-specific methylation defect of development-related enhancers during embryonic development in Dnmt3a2^−/−^ mice likely increases the chances of stochastic developmental defects observed in a subset of animals.

### Dnmt3a2 loss reduces the methylation of infertility-related imprinted genes in sperm.

**Figure 4:**
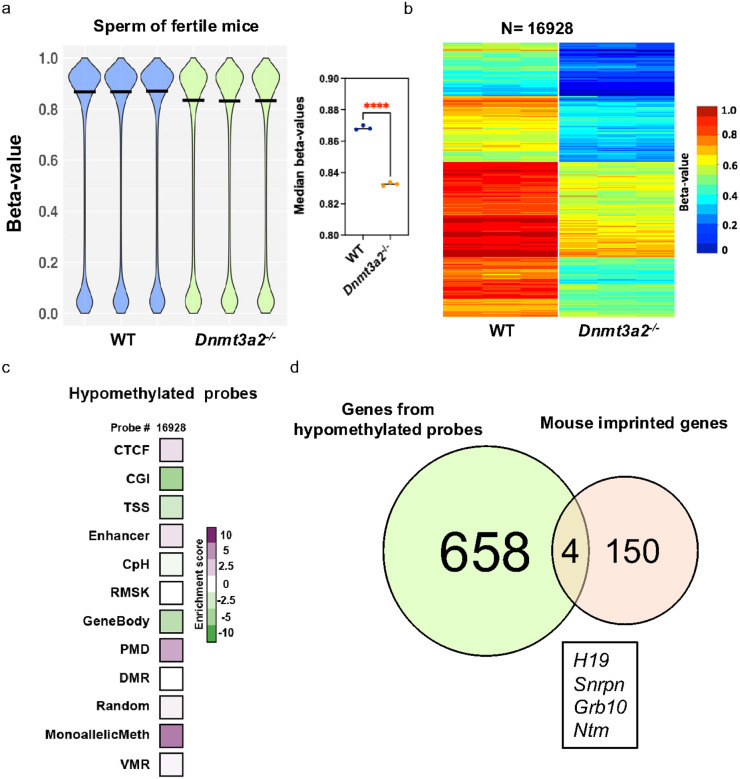
Dnmt3a2 loss reduces the methylation of infertility-related imprinted genes in mouse sperm cells. **a,** DNA methylation violin plot showing the distribution of the β-values of the autosomal probes in the Infinium mouse EPIC array. β-values range from 0.0 to 1.0 for each probe, indicating total lack of methylation or complete methylation, respectively. The right panel compares the median beta-value for the WT to the *Dnmt3a2*^−/−^ sperm. ****, P < 0.0001 by two-tailed unpaired t test. **b,** Heatmap showing hypomethylated CpG sites in *Dnmt3a2*^−/−^ mice compared to WT. Hypomethylated probes were selected using a cutoff of beta-value difference greater than 0.2 and p < 0.05 by two-tailed unpaired t test. Methylation levels are represented by a cold to warm color scale (β-value 0–1, 0 – 100% methylated), where every row represents an individual probe, and every column represents sperm from an individual mouse. **c,** Heatmap depicting the enrichment score of hypomethylated probes (DMPs) for multiple genomic elements included in the Infinium EPIC array. The classified genomic element are as follows: CTCF binding sites (CTCF), CpG island (CGI), transcription start site (TSS), enhancer (Enhancer), CpH methylation (CPH), repetitive elements (RMSK), gene bodies (GeneBody), partially methylated domains (PMD), differentially methylated regions (DMR), random location probes (Random), monoallelic methylation, including the ICRs of imprinted genes (MonoallelicMeth), and metastable alleles and variably methylated regions (VMRs). **d,** Venn diagram depicting the intersection between genes from monoallelic hypomethylated DMPs identified by the EPIC array and mouse imprinted genes.

Considering that male infertility was the most frequent phenotype in *Dnmt3a2*^−/−^ mice (Table 1), and that the expression of this isoform remains present in adult mice testes^22^, we hypothesized that the loss of Dntm3a2 might affect the DNA methylation profile in mouse sperm. For the four infertile *Dnmt3a2*^−/−^ mice, we failed to isolate any motile sperm from the one example we examined. We therefore isolated motile sperm from fertile WT and *Dnmt3a2*^−/−^ mice and evaluated the DNA methylation patterns for all the autosomal probes using the EPIC array. Using a beta-value cutoff of ≥ 0.2 and p < 0.05, we found that *Dnmt3a2*^−/−^ sperm showed significant hypomethylation (6.5% of total), (Fig. 4a, b). The hypomethylated probes were enriched in four genomic elements: Monoallelic methylation (5.9%), PMD (4.0%), CTCF (1.6%), and Enhancer (1.4%) (Fig. 4c), indicating that Dnmt3a2 is required for complete DNA methylation of multiple regions in spermatozoa.

Imprinted genes are known to play a critical role in spermatogenesis, and alteration in their methylation can impede normal spermatogenesis^28–30^. Considering the importance of Dnmt3a2’s activity on monoallelic methylation in the process of gene imprinting (Fig. 3a), we further investigated if imprinted genes were hypomethylated in sperm from *Dnmt3a2*^−/−^ mice. We identified 1498 hypomethylated probes distributed over 658 genes in the monoallelic methylation category (Fig. 4d). We compared the hypomethylated genes against a database of 150 previously described mouse imprinted genes and found 4 hypomethylated imprinted genes in *Dnmt3a2*^−/−^ sperm: *H19, Snrpn, Grb10* and *Ntm* (Fig. 4d). These data indicates that *Dnmt3a2*^−/−^ mice display a gene imprinting defect in sperm. Interestingly, DNA methylation defects of *H19* and *Snrpn* have been previously associated with male infertility^30^, suggesting that Dnmt3a2 expression is necessary for proper methylation of imprinted genes associated with sperm development and fertility.

### Dnmt3a2 KO-induced methylation loss in sperm DNA follows a stochastic pattern.

**Figure 5:**
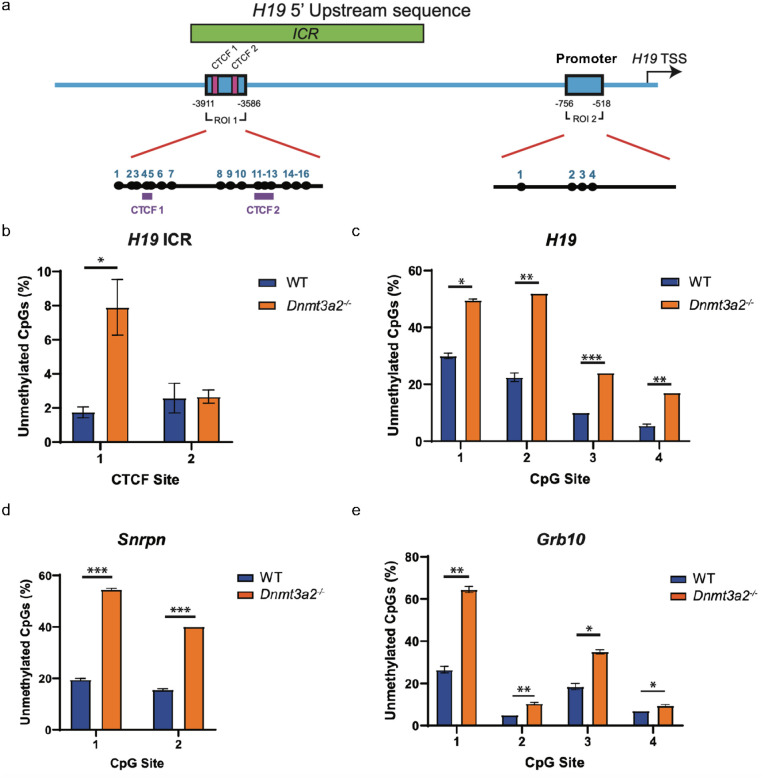
Dnmt3a2 loss induces CTCF binding site loss of methylation at the *H19* ICR. **a,** Schematic representation of the *H19* 5’ Upstream region. The studied regions of interest (ROI 1 and ROI 2, blue box) are located inside the ICR (Green box) encompassing two CTCF binding sites (magenta boxes), and at the promoter region of *H19*, respectively. **b-e,** Graph bar depicting the percentage of unmethylated CpGs in the *H19* ICR CTCF binding sites (ROI1) (**b**), *H19* promoter (ROI2)(**c**), *Snrpn* enhancer (**d**), and ICR of *Grb10* (**e**) from WT and *Dnmt3a2*^−/−^ sperm DNA (n= 4–5; mean ± SEM; *, p < 0.05; **, p < 0.01 and ***, p <0.001 by two-tailed unpaired t tests).

We examined the hypomethylated regions of three imprinted genes using targeted amplicon bisulfite sequencing to include adjacent CpG sites not present in the array probes. For the *H19* gene, we confirmed that *Dnmt3a2*^−/−^ sperm showed greater hypomethylation of the CTCF1 (8%) compared to WT (2%), but not the CTCF2 binding site of the ICR (Fig. 5a, 5b and Extended Data Fig. 4). Similarly, the region located at the promoter of *H19* showed significant hypomethylation of the 4 CpG sites evaluated (Fig. 5a, 5c and Extended Data Fig. 5). In the *Snrpn*, and *Grb10*, each CpG site evaluated showed a different degree of hypomethylation compared to the controls. There was a 2-to-3-fold change increase in the level of hypomethylation for most sites (Fig. 5d, 5d, Extended Data Figs. 6 and 7). Next, we evaluated the DNA methylation patterns of these regions in individual DNA sequences and observed that DNA methylation loss did not follow a specific pattern (Extended Data Fig. 4–7). The number of unmethylated CpG sites per sequence was variable, and they appeared randomly distributed within the sequence. Additionally, our data shows that *Dnmt3a2*^−/−^ mice exhibit DNA methylation defects to be stochastically distributed within the sperm population, as evidenced by the multiple unique methylation patterns shown in the DNA sequences evaluated (Fig. S4 to S7). Previous reports have confirmed the expression of Dnmt3a2 isoform during the latter stages of sperm maturation^31^. Our data further supports the need for Dnmt3a2 expression during spermatogenesis, suggesting an active role for Dnmt3a2 at completing the methylation of multiple genomic regions in sperm, more importantly in imprinted genes related to male infertility, such as *H19*.

## Discussion

Our findings highlight a developmentally regulated interplay between the Dnmt3a1 and Dnmt3a2 isoforms, Dnmt3a2 plays a central role in securing proper enhancer, CTCF sites and imprinted gene methylation during embryogenesis, while Dnmt3a1 focuses on securing proper enhancer methylation in the postnatal stage of development. The compensation of methylation deficits after birth by Dnmt3a1 suggests a division of labor between the isoforms, with Dnmt3a2 acting as the primary *de novo* methyltransferase during early development and Dnmt3a1 completing DNA methylation patterns postnatally. The increased incidence of sporadic phenotypes in Dnmt3a2-deficient mice underscores the potential importance of proper enhancer methylation in ensuring robust developmental outcomes. This study provides new insights into the isoform-specific functions of Dnmt3a and emphasizes the need to consider these distinct roles when investigating the mechanisms underlying functional elements, such as enhancers, CTCF sites and ICRs, and developmental disorders.

The distinct functional roles of Dnmt3a isoforms during development cannot be fully explained by their different expression patterns alone. While our data demonstrate equivalent expression levels of Dnmt3a1 and Dnmt3a2 during embryogenesis in WT mice (Fig. 1b, c), their knockout models reveal different impacts on DNA methylation. Specifically, *Dnmt3a2*^−/−^ embryos exhibit significant hypomethylation at E15.5, whereas *Dnmt3a1*^−/−^ embryos show negligible methylation defects at this stage (Table 1). This suggests that Dnmt3a2 is the dominant isoform responsible for establishing *de novo* methylation during early embryonic development, likely through its collaboration with Dnmt3L to target enhancers and CTCF-binding sites, as well as imprinted loci in the parental gametes^16,32,33^. In contrast, our data also demonstrates that Dnmt3a1 becomes indispensable postnatally, particularly at bivalent promoters and enhancers, where its loss provides the explanation of severe developmental abnormalities^22^. Our findings underscore a division of labor between the two Dnmt3a isoforms and highlights the subtle regulation of DNA methylation landscapes necessary for robust developmental outcomes.

The sporadic phenotypes observed in *Dnmt3a2*^−/−^ mice further underscore the importance of methylation fidelity during embryogenesis. The incomplete penetrance of developmental defects in these mice suggests that Dnmt3a2-mediated methylation at enhancers acts as a buffer against intrinsic developmental heterogeneity—a phenomenon that is stochastic in nature^26,34,35^—thereby promoting robust developmental outcomes and limiting phenotypic variability. This is supported by our methylation analysis of spermatozoa from fertile *Dnmt3a2*^−/−^mice, which revealed stochastic hypomethylation at ICRs, promoters and enhancers of imprinted genes compared to WT controls (Fig. 5 and Extended Data Fig. 4–7), potentially explaining the observed sporadic cases of male infertility^16,28–30^. While bulk methylation assays in E15.5 brain and liver tissues (using array-based approaches) reflect averaged methylation levels across heterogeneous cell populations (Fig. 2), we can infer that Dnmt3a2 loss results in cell-to-cell variability of DNA methylation at enhancers during embryogenesis. Such stochastic hypomethylation likely disrupts the precision of transcriptional regulation in individual cells, ultimately manifesting as sporadic developmental phenotypes in *Dnmt3a2*^−/−^mice. Taken together, the loss of Dnmt3a2-mediated epigenetic regulation during embryogenesis appears to destabilize developmental processes, increasing the possibility of subsequent abnormalities.

The sporadic phenotypes observed in *Dnmt3a2*^−/−^mice, including anophthalmia, hydrocephalus, and hydronephrosis, which reflect profound developmental defects, such as the complete absence of the ureter (hydronephrosis), eye (anophthalmia), and potentially ciliary dysfunction (hydrocephalus), can be attributed to DNA methylation changes caused by the loss of Dnmt3a2 during critical developmental windows, rather than genetic mutations or environmental causes. For example, hydronephrosis in inbred C57BL/6 mice has been linked to autosomal recessive mutations in *Aqp2cph*, which induce nephrogenic diabetes insipidus and polyuria, leading to obstructive nephropathy without structural deficits in the pyeloureteral peristaltic machinery^36–38^. In such models, hydronephrosis arises postnatally due to functional urinary tract obstruction rather than developmental absence of the ureter. By contrast, the complete agenesis of the ureter in *Dnmt3a2*^−/−^mice implicates a failure in early developmental patterning, likely stemming from disrupted epigenetic regulation during critical morphogenetic windows. In addition, the incidence of hydrocephalus in *Dnmt3a2*^−/−^ mice was 1% (2 out of 203), a 34-fold increase compared to the background level of 0.029% in the inbred C57BL/6J strain (https://www.jax.org/news-and-insights/2003/july/hydrocephalus-in-laboratory-mice), strongly implicating Dnmt3a2 loss as a contributing factor. Similarly, the observed eye abnormalities, which may result from defects in lens development, and the severe hydronephrosis phenotype, which cannot be explained by genetic mutations alone, further support the role of epigenetic dysregulation in these defects. The fact that environmental factors like alcohol and other teratogens can exacerbate the rate of developmental defects suggests that epigenetic mechanisms, in addition to genetic factors, play a critical role in shaping developmental outcomes. Future studies mapping methylation dynamics at single-cell resolution in *Dnmt3a2*^−/−^ embryonic tissues could directly test whether stochastic or uniform hypomethylation at lineage-specific enhancers underlies these phenotypes.

In summary, this study provides new insights into the isoform-specific functions of Dnmt3a and highlights the importance of considering these distinct roles when investigating the epigenetic regulation of development and disease. The observed phenotypes in Dnmt3a2-deficient mice emphasize the potential consequences of enhancer dysregulation and suggest that Dnmt3a2 may serve as a safeguard against developmental instability. Future studies should explore the molecular mechanisms underlying the isoform-specific functions of Dnmt3a and their potential implications for human developmental disorders and diseases linked to epigenetic dysregulation. Overall, this work advances our understanding of the complex roles of Dnmt3a isoforms in shaping the epigenetic landscape and ensuring proper development.

## Methods

### Animals and husbandry

This research complies with ethical regulations, with protocols approved by the Institutional Animal Care and Use Committee (Van Andel Institute (VAI); protocols 20-06-006 and 23-06-013).

The *Dnmt3a1* and *Dnmt3a2* KO mice were generated by injecting CRISPR–Cas9/sgRNA mixtures into B6C3F2 zygotes and were performed by the VAI Transgenics Core (RRID:SCR_022914). Briefly, 50 ng/ul HiFi AltR Cas9 (Integrated DNA Technologies), 25 ng/ul crRNA:tracrRNA1 and 25 ng/ul crRNA:tracrRNA2 (Integrated DNA Technologies) mixed in modified Tris-EDTA (TE) buffer (10mM Tris-HCl pH 7.4, 0.1mM EDTA) was injected into B6C3F2 zygote cytoplasm. Sequences of gRNA are listed in Supplementary Table 1. Mosaic mice from injections were then back crossed to WT C57BL/6J mice for at least seven generations to obtain heterozygous mice. The homozygous mice were generated by intercrossing the heterozygotes. Littermate controls were used for all comparisons between WT and mutants. For genotyping, ear punch biopsies were collected and the primers used are listed in Supplementary Table 1. All mice were housed at VAI Vivarium core (RRID:SCR_023211) in individual ventilated cages (Tecniplast, Sealsafe Plus GM500 in DGM Racks) with a 12-h/12-h light/dark cycle at 22 ± 1 °C and 30%–70% humidity, and were fed breeder chow (LabDiet, 5021, 0006540). Mice were checked daily by animal keepers and two to three times per week by expert VAI Vivarium Core Staff for health, well-being and any health concerns. Mice with reported phenotypes were euthanized via CO2 asphyxiation.

### Tissue lysate preparation and western blot analysis

To examine Dnmt3a1 and Dnmt3a2 expression, brain tissue from E14.5 embryos were crushed and lysed in 200 μl T-PER^™^ Tissue Protein Extraction Reagent (Thermo Scientific) supplemented with 2% SDS and 1× protease inhibitors (Roche). The extracted proteins were denatured in SDS-loading buffer and analyzed by western blot using DNMT3A (E9P2F) Rabbit mAb (Cell Signaling Technology CST 49768) and b-tublin (D3U1W) Mouse mAb (Cell Signaling Technology CST-86298).

### Tissue preparation and DNA extraction

Brain (right cerebrum) and liver from E15.5 embryo and PD21 mice were digested in lysis buffer (10mM Tris-HCl pH 8.0, 10mM EDTA, 1% SDS, and 2 mg/ml Proteinase K) overnight at 55°C, followed by phenol-chloroform 1:1 isolation and 50% isopropyl alcohol precipitation. DNA pellet was then washed twice in 70% ethanol and dissolved in TE buffer (10mM Tris-HCl pH 8.0, 0.1mM EDTA).

### DNA isolation from fertile mice sperm

Cauda epididymis from WT and fertile *Dnmt3a2*^−/−^mice were removed and cleaned before suspending them in M16 buffer (95mM NaCl; 4.8mM KCl; 1.1mM KH2PO4, 1.2mM MgSO4, 0.15% Sodium lactate; 5.5mM Glucose, 2.1mg/ml NaHCO3, 0.36mg/ml Sodium pyruvate, 2.3mM CaCl2, and 4mg/ml BSA). Cauda epididymis was nicked, transferred to a tube, and incubated in M16 buffer for 2 hours in an incubator (37°C, 5% CO2) to allow healthy sperm to swim up. Supernatant containing sperm was removed without disrupting the bottom and sperm spun down by centrifugation (3000 rpm, 6 min). Sperm was digested in lysis buffer (10mM Tris-HCl pH 8.0, 10mM EDTA, 1% SDS, 0.1M DTT and 2 mg/ml Proteinase K) overnight at 55°C, followed by phenol-chloroform 1:1 isolation and 100% ethanol precipitation. DNA pellet was then washed twice in 70% ethanol and dissolved in TE buffer (10mM Tris-HCl pH 8.0, 0.1mM EDTA).

### Mouse DNA methylation array

DNA samples (20–500 ng) were bisulfite converted using the Zymo EZ DNA Methylation Kit (Zymo Research) following the manufacturer’s protocol with modifications for the Illumina Infinium methylation assays. DNA methylation was assessed using Illumina Infinium Mouse Methylation BeadChip array (MM285), conducted by the VAI Genomics core (RRID:SCR_022913) following the manufacturer’s specifications. Arrays were scanned on the Illumina iScan platform, and probe-specific calls were made using Illumina GenomeStudio version 2011.1 to generate IDAT files.

### DNA methylation analysis

Quality control, preprocessing and normalization process were performed using the SeSAMe pipeline (version 1.24.0) on Bioconductor^39^, and the methylation status of individual CpG site was reported as a b-value, ranging from 0 (unmethylated) to 1 (fully methylated). Probes located on the sex chromosomes were excluded from the analysis to enable comparison between KO and WT tissues independent of sex. Probe enrichment analysis was performed using the SeSAMe knowYourCG module^39^, with annotations based on the knowYourCG tool, and related information available at http://zwdzwd.github.io/InfiniumAnnotation#mouse. The ChromHMM annotation was derived from a mouse consensus by the ENCODE project profiling 66 mouse epigenomes across 12 tissues at daily intervals from embryonic day 11.5 to birth^40^. Gene ontology analysis of probe-enriched genes was performed using the Database for Annotation, Visualization, and Integrated Discovery (DAVID) functional annotation clustering (v2023q4)^41^. Further data visualization of SeSAMe output was performed using RStudio (2024.12.1.563) in R (version 4.4.2).

### Nucleoside Quantitation in DNA digests by LC-MS

Absolute nucleoside quantitation in DNA digests was accomplished using liquid chromatography (LC)-mass spectrometry (MS) in the Van Andel Institute Mass Spectrometry Core (RRID:SCR_024903). An external calibration curve was prepared from a stock mix containing 2’deoxycytidine (dC; 100 μg/mL) (30125, Caymen Chemical), 5-methyl-2’deoxycytidine (5mdC; 4 μg/mL) (16166, Caymen Chemical), and 2’deoxyguanosine (dG; 100 μg/mL) (9002864, Caymen Chemical). The stock mix underwent a 7-step serial dilution of half-log steps for eight total curve points. 35μL of DNA digests and standard curve samples were extracted with 315μL of 80% methanol (v/v) containing [13C515N]2’deoxycytidine (6.3 ng/mL) (D239552, Toronto Research Chemicals), [D3]5-methyl-2’deoxycytidine (12.6ng/mL) (M295902, Toronto Research Chemicals), and [13C5] 2’deoxyguanosine (12.6ng/mL) (D232617, Toronto Research Chemicals) as internal standards.

Samples and standards were analyzed with an Agilent 6470 triple quadrupole mass spectrometer coupled with an Agilent ultra-high performance liquid chromatography 1290 Infinity II. 2 μL of each sample was injected separated using a 21-minute gradient on a Cortecs T3 Column (1.6 μm, 2.1mm × 150mm, 186008500, Waters, Eschborn, Germany) combined with a Cortecs VanGuard cartridge (1.6 μm, 2.1 mm × 5 mm, 186008508, Waters). Mobile phase A consisted of LC/MS grade water (W6, Fisher) with 1mM ammonium acetate (73594, Sigma), and 0.01% ammonium hydroxide (A470, Fisher). Mobile phase B consisted of 99% Acetonitrile (A955–4, Fisher) and 1% LC/MS grade water (W6, Fisher). Column temperature was kept at 30 °C, flow rate was held at 0.3 mL/min, and the chromatography gradient was as follows: 0–6 min held at 0% B, 6–9 min ramp from 0 to 10% B, 9–14 min ramp from 10% to 50% B, 14 to 18.9 min ramp from 50% to 99% B. At 19 min, flow is changed to 0% B at 0.4 ml/min, and held until 20.5 min, then decreased to 0.3 ml/min by 21 min. Mass spectrometer parameters were: Gas flow at 13 L/min at 80°C, sheath gas flow at 11 L/min at 275 °C and the nebulizer was set to 30 psi. Capillary voltage was +2500 and nozzle voltage was +500. Data were acquired using dynamic multiple reaction monitoring (dMRM) including at least two transition per compound. The transition list was developed and optimized using neat analytical standards and parameters are provided in Supplemental Table 2. The dMRM parameters were determined based on running LC/MS grade analytical standards for each target compound. Peak picking and integration was performed using Skyline Software (v 24.1). The ISTD peak area was used to correct for matrix effect differences between the samples and standards. The corrected peak areas were used to generate a linear or quadratic regression for quantitation of the target analytes. The quantified analytes (ng/mL) were then used to calculate 5mdC/dG ratio.

### Targeted amplicon bisulfite sequencing and analysis

Bisulfite conversion of DNA was carried out using the EZ DNA Methylation-Gold Kit (Zymo Research, #D5006) according to manufacturer’s recommendations. *H19* ICR region was amplified using a nested PCR approach. Briefly, 100ng of bisulfite-converted DNA was amplified with primer set 1 using ZymoTaq Polymerase (Zymo Research, #E2002) under the following amplification conditions: 95°C for 10 minutes, 35 cycles of amplification (95°C for 30 seconds, 54°C for 30 seconds, 72°C for 40 seconds), final extension at 72°C for 7 minutes. Second round of amplification was carried out using template from first reaction with the primer set 2 using same amplification conditions. *H19, Snrpn*, and *Grb10* regions were amplified using MyTaq Red Mix (Meridian Biosciences, BIO-25044) under the following amplification conditions: 95°C for 2 minutes, 40 cycles of amplification (95°C for 30 seconds, 58°C for 30 seconds, 72°C for 60 seconds), final extension at 72°C for 5 minutes. Primers used are listed in supplementary table 1. Bisulfite PCR products were sequenced using the Genewiz Amplicon-EZ sequencing service (Azenta Life Sciences) and aligned to reference sequences using the BISulfite-seq CUI Toolkit^42^. BAM files from sorted sequences were created using SAMtools^43^ and loaded in IGV 2.17.4 for visualization and calculation of CpG dinucleotide methylation percentage.

### Statistical analysis

Experimental data were analyzed using Prism 10 software (GraphPad, version 10.4.1) by one-way ANOVA, two-tailed Student’s t-test or indicated in the corresponding figure legends. Statistical significance levels are denoted as follows: *,p < 0.05; **, p < 0.01; ***, p < 0.001; ****, p < 0.0001, ns, not significant. Data are presented as median ± 95%CI or mean ± s.e.m. as indicated in the corresponding figure legends. No statistical methods were used to predetermine sample size.

## Supplementary Material

Supplementary Files

This is a list of supplementary files associated with this preprint. Click to download.

- Tables.pdf
- Supplementaryinformation.docx
- nrreportingsummaryPJones061625.pdf
- Extenddata.pdf

## Data Availability

Data generated for this work have been deposited to GEO under accession number GSE295720 with the reviewer’s token yfsngyyinxabdmj.

## Code availability

All publicly available codes and tools used to analyze the data are reported and referenced in the Methods. Any additional information required to reanalyze the data reported in this paper is available from the lead contact upon request.

## References

[1] Jones P.A.: Functions of DNA methylation: islands, start sites, gene bodies and beyond. Nat. Rev. Genet. 13, 484–492 (2012). 10.1038/nrg3230 22641018

[2] Ong C.T., Corces V.G.: Enhancer function: new insights into the regulation of tissue-specific gene expression. Nat. Rev. Genet. 12, 283–293 (2011). 10.1038/nrg2957 21358745 PMC3175006

[3] Schuff M., Strong A.D., Welborn L.K., Ziermann-Canabarro J.M.: Imprinting as Basis for Complex Evolutionary Novelties in Eutherians. Biology (Basel). 13 (2024). 10.3390/biology13090682 PMC11428813 39336109

[4] Gifford C.A., : Transcriptional and epigenetic dynamics during specification of human embryonic stem cells. Cell. 153, 1149–1163 (2013). 10.1016/j.cell.2013.04.037 23664763 PMC3709577

[5] Xie W., : Epigenomic analysis of multilineage differentiation of human embryonic stem cells. Cell. 153, 1134–1148 (2013). 10.1016/j.cell.2013.04.022 23664764 PMC3786220

[6] Okano M., Bell D.W., Haber D.A., Li E.: DNA methyltransferases Dnmt3a and Dnmt3b are essential for de novo methylation and mammalian development. Cell. 99, 247–257 (1999). 10.1016/s0092-8674(00)81656-6 10555141

[7] Bick A.G., : Inherited causes of clonal haematopoiesis in 97,691 whole genomes. Nature. 586, 763–768 (2020). 10.1038/s41586-020-2819-2 33057201 PMC7944936

[8] Jaiswal S., : Age-related clonal hematopoiesis associated with adverse outcomes. N Engl. J. Med. 371, 2488–2498 (2014). 10.1056/NEJMoa1408617 25426837 PMC4306669

[9] Ley T.J., : DNMT3A mutations in acute myeloid leukemia. N Engl. J. Med. 363, 2424–2433 (2010). 10.1056/NEJMoa1005143 21067377 PMC3201818

[10] Xie M., : Age-related mutations associated with clonal hematopoietic expansion and malignancies. Nat. Med. 20, 1472–1478 (2014). 10.1038/nm.3733 25326804 PMC4313872

[11] Yang L., Rau R., Goodell M.A.: DNMT3A in haematological malignancies. Nat. Rev. Cancer. 15, 152–165 (2015). 10.1038/nrc3895 25693834 PMC5814392

[12] Tatton-Brown K., : Mutations in the DNA methyltransferase gene DNMT3A cause an overgrowth syndrome with intellectual disability. Nat. Genet. 46, 385–388 (2014). 10.1038/ng.2917 24614070 PMC3981653

[13] Heyn P., : Gain-of-function DNMT3A mutations cause microcephalic dwarfism and hypermethylation of Polycomb-regulated regions. Nat. Genet. 51, 96–105 (2019). 10.1038/s41588-018-0274-x 30478443 PMC6520989

[14] Christian D.L., : DNMT3A Haploinsufficiency Results in Behavioral Deficits and Global Epigenomic Dysregulation Shared across Neurodevelopmental Disorders. Cell. Rep. 33, 108416 (2020). 10.1016/j.celrep.2020.108416 33238114 PMC7716597

[15] Tovy A., : Constitutive loss of DNMT3A causes morbid obesity through misregulation of adipogenesis. Elife. 11 (2022). 10.7554/eLife.72359 PMC9150890 35635747

[16] Kaneda M., : Essential role for de novo DNA methyltransferase Dnmt3a in paternal and maternal imprinting. Nature. 429, 900–903 (2004). 10.1038/nature02633 15215868

[17] Challen G.A., : Dnmt3a is essential for hematopoietic stem cell differentiation. Nat. Genet. 44, 23–31 (2011). 10.1038/ng.1009 22138693 PMC3637952

[18] Wu Z., : Dnmt3a regulates both proliferation and differentiation of mouse neural stem cells. J. Neurosci. Res. 90, 1883–1891 (2012). 10.1002/jnr.23077 22714992 PMC3418436

[19] Nguyen S., Meletis K., Fu D., Jhaveri S., Jaenisch R.: Ablation of de novo DNA methyltransferase Dnmt3a in the nervous system leads to neuromuscular defects and shortened lifespan. Dev. Dyn. 236, 1663–1676 (2007). 10.1002/dvdy.21176 17477386

[20] Gao Q., : Deletion of the de novo DNA methyltransferase Dnmt3a promotes lung tumor progression. Proc. Natl. Acad. Sci. U S A. 108, 18061–18066 (2011). 10.1073/pnas.1114946108 22011581 PMC3207684

[21] Chen T., Ueda Y., Xie S., Li E.: A novel Dnmt3a isoform produced from an alternative promoter localizes to euchromatin and its expression correlates with active de novo methylation. J. Biol. Chem. 277, 38746–38754 (2002). 10.1074/jbc.M205312200 12138111

[22] Gu T., : The disordered N-terminal domain of DNMT3A recognizes H2AK119ub and is required for postnatal development. Nat. Genet. 54, 625–636 (2022). 10.1038/s41588-022-01063-6 35534561 PMC9295050

[23] Yang L., : DNMT3A Loss Drives Enhancer Hypomethylation in FLT3-ITD-Associated Leukemias. Cancer Cell. 29, 922–934 (2016). 10.1016/j.ccell.2016.05.003 27300438 PMC4908977

[24] Ma P., de Waal E., Weaver J.R., Bartolomei M.S., Schultz R.M.: A DNMT3A2-HDAC2 Complex Is Essential for Genomic Imprinting and Genome Integrity in Mouse Oocytes. Cell. Rep. 13, 1552–1560 (2015). 10.1016/j.celrep.2015.10.031 26586441 PMC4662907

[25] Thakur A., : Widespread recovery of methylation at gametic imprints in hypomethylated mouse stem cells following rescue with DNMT3A2. Epigenetics Chromatin. 9, 53 (2016). 10.1186/s13072-016-0104-2 27895716 PMC5118886

[26] Whitelaw N.C., : Reduced levels of two modifiers of epigenetic gene silencing, Dnmt3a and Trim28, cause increased phenotypic noise. Genome Biol. 11, R111 (2010). 10.1186/gb-2010-11-11-r111 21092094 PMC3156950

[27] Zhou W., : DNA methylation dynamics and dysregulation delineated by high-throughput profiling in the mouse. Cell. Genom. 2 (2022). 10.1016/j.xgen.2022.100144 PMC9306256 35873672

[28] Santi D., De Vincentis S., Magnani E., Spaggiari G.: Impairment of sperm DNA methylation in male infertility: a meta-analytic study. Andrology. 5, 695–703 (2017). 10.1111/andr.12379 28718528

[29] Asenius F., Danson A.F., Marzi S.J.: DNA methylation in human sperm: a systematic review. Hum. Reprod. Update. 26, 841–873 (2020). 10.1093/humupd/dmaa025 32790874

[30] Cannarella R., : H19 Sperm Methylation in Male Infertility: A Systematic Review and Meta-Analysis. Int. J. Mol. Sci. 24 (2023). 10.3390/ijms24087224 PMC10139270 37108386

[31] La Salle S., Trasler J.M.: Dynamic expression of DNMT3a and DNMT3b isoforms during male germ cell development in the mouse. Dev. Biol. 296, 71–82 (2006). 10.1016/j.ydbio.2006.04.436 16725135

[32] Bourc’his D., Xu G.L., Lin C.S., Bollman B., Bestor T.H.: Dnmt3L and the establishment of maternal genomic imprints. Science. 294, 2536–2539 (2001). 10.1126/science.1065848 11719692

[33] Hata K., Okano M., Lei H., Li E.: Dnmt3L cooperates with the Dnmt3 family of de novo DNA methyltransferases to establish maternal imprints in mice. Development. 129, 1983–1993 (2002). 10.1242/dev.129.8.1983 11934864

[34] Dalgaard K., : Trim28 Haploinsufficiency Triggers Bi-stable Epigenetic Obesity. Cell. 164, 353–364 (2016). 10.1016/j.cell.2015.12.025 26824653 PMC4735019

[35] Castillo-Fernandez J.E., Spector T.D., Bell J.T.: Epigenetics of discordant monozygotic twins: implications for disease. Genome Med. 6, 60 (2014). 10.1186/s13073-014-0060-z 25484923 PMC4254430

[36] Taylor D.M., Fraser H.: Hydronephrosis in inbred strains of mice with particular reference to the BRVR strain. Lab. Anim. 7, 229–236 (1973). 10.1258/002367773780944067 4747258

[37] Horton C.E. Jr., : Congenital progressive hydronephrosis in mice: a new recessive mutation. J. Urol. 140, 1310–1315 (1988). 10.1016/s0022-5347(17)42033-7 3184310

[38] McDill B.W., Li S.Z., Kovach P.A., Ding L., Chen F.: Congenital progressive hydronephrosis (cph) is caused by an S256L mutation in aquaporin-2 that affects its phosphorylation and apical membrane accumulation. Proc. Natl. Acad. Sci. U S A. 103, 6952–6957 (2006). 10.1073/pnas.0602087103 16641094 PMC1459000

[39] Zhou W., Triche T.J. Jr., Laird P.W., Shen H.: SeSAMe: reducing artifactual detection of DNA methylation by Infinium BeadChips in genomic deletions. Nucleic Acids Res. 46, e123 (2018). 10.1093/nar/gky691 30085201 PMC6237738

[40] van der Velde A., : Annotation of chromatin states in 66 complete mouse epigenomes during development. Commun. Biol. 4, 239 (2021). 10.1038/s42003-021-01756-4 33619351 PMC7900196

[41] Sherman B.T., : DAVID: a web server for functional enrichment analysis and functional annotation of gene lists (2021 update). Nucleic Acids Res. 50, W216–W221 (2022). 10.1093/nar/gkac194 35325185 PMC9252805

[42] Zhou W., : BISCUIT: an efficient, standards-compliant tool suite for simultaneous genetic and epigenetic inference in bulk and single-cell studies. Nucleic Acids Res. 52, e32 (2024). 10.1093/nar/gkae097 38412294 PMC11014253

[43] Li H., : The Sequence Alignment/Map format and SAMtools. Bioinformatics. 25, 2078–2079 (2009). 10.1093/bioinformatics/btp352 19505943 PMC2723002

